# Lung Cancer Attributable to Indoor Radon Exposure in France: Impact of the Risk Models and Uncertainty Analysis

**DOI:** 10.1289/ehp.9070

**Published:** 2006-05-30

**Authors:** Olivier Catelinois, Agnès Rogel, Dominique Laurier, Solenne Billon, Denis Hemon, Pierre Verger, Margot Tirmarche

**Affiliations:** 1 Institute for Radiation Protection and Nuclear Safety, Fontenay-aux-Roses, France; 2 French Institute of Health and Medical Research, Villejuif, France; 3 Regional Health Observatory Provence Alpes Côte d’Azur, Marseille, France

**Keywords:** lung cancer, radiation, radon, risk assessment, uncertainty analysis

## Abstract

**Objective:**

The inhalation of radon, a well-established human carcinogen, is the principal—and omnipresent—source of radioactivity exposure for the general population of most countries. Scientists have thus sought to assess the lung cancer risk associated with indoor radon. Our aim here is to assess this risk in France, using all available epidemiologic results and performing an uncertainty analysis.

**Methods:**

We examined the exposure–response relations derived from cohorts of miners and from joint analyses of residential case-control studies and considered the interaction between radon and tobacco. The exposure data come from measurement campaigns conducted since the beginning of the 1980s by the Institute for Radiation Protection and Nuclear Safety and the Directorate-General of Health in France. We quantified the uncertainties associated with risk coefficients and exposures and calculated their impact on risk estimates.

**Results:**

The estimated number of lung cancer deaths attributable to indoor radon exposure ranges from 543 [90% uncertainty interval (UI), 75–1,097] to 3,108 (90% UI, 2,996–3,221), depending on the model considered. This calculation suggests that from 2.2% (90% UI, 0.3–4.4) to 12.4% (90% UI, 11.9–12.8) of these deaths in France may be attributable to indoor radon.

**Discussion:**

In this original work we used different exposure–response relations from several epidemiologic studies and found that regardless of the relation chosen, the number of lung cancer deaths attributable to indoor radon appears relatively stable. Smokers can reduce their risk not only by reducing their indoor radon concentration but also by giving up smoking.

Radon is a chemically inert radioactive gas of natural origin, produced by the disintegration of uranium and radium located in the earth’s crust. Radon exposure, at various levels, is omnipresent for the general public. Radon inhalation is the main source of exposure to radioactivity for most people throughout the world [National Research Council’s (NRC) Committee on the Biological Effects of Ionizing Radiation (BEIR) 1999; National Council for Radiation Protection and Measurements 1984a, 1984b; U.S. Environmental Protection Agency (EPA) 2003; United Nations Scientific Committee on the Effects of Atomic Radiation (UNSCEAR) 2000]. Most inhaled radon is rapidly exhaled, but the inhaled decay products—readily deposited in the lung epithelium—irradiate sensitive cells in the airways and thereby enhance the risk of lung cancer. In 1988, the International Agency for Research on Cancer (IARC) declared radon to be carcinogenic for humans (lung cancer) and classified as a group 1 carcinogen (IARC 1988), based on the results of experimental animal and epidemiologic studies, in particular among uranium miners. In 1988, the available results came from studies of high exposure levels. Extrapolation of this risk to the general population, who are exposed to lower levels in residential settings, raised numerous questions.

In recent years, the average annual exposure of uranium miners has fallen to levels similar to the concentrations inhaled in some homes, and discussion today focuses on the transposition of the risk from occupational to general populations. Miners are almost all adult males, exposed in conditions different from residential exposure: they perform substantial amounts of heavy labor in an atmosphere polluted by dust and fumes. Several case-control studies of residential radon have tested the validity of this risk transposition in the past decade (Auvinen et al. 1996; Kreienbrock et al. 2001; Letourneau et al. 1994; Schoenberg et al. 1990), but lack of statistical power prevented most of them from showing a significant risk. To deal with this problem, several joint analyses have been conducted in recent years. They report a significant lung cancer risk after domestic radon exposure (Darby et al. 2005; Krewski et al. 2005; Lubin 2003).

Scientists have used all of these data to assess the lung cancer risk associated with indoor radon. The principal risk assessments come from the United States, the United Kingdom, and Canada (BEIR 1999; Darby et al. 2005; Krewski et al. 2005). These studies have two principal methodologic problems. The first is related to the choice of the exposure–response relation and its use in the context of general population exposure. Past risk assessment studies generally used exposure–response relations derived from miners’ cohorts as recommended by BEIR (1990, 1999). The second methodologic problem involves the role of the uncertainty analysis in the risk assessment process. Uncertainty analysis is an essential step that is too seldom performed.

The aim of this study was to assess the lung cancer risk associated with indoor radon exposure in France on the basis of French measurements and the major epidemiologic results available. Specifically, we applied several different exposure–response relations obtained from miners’ cohorts and from joint analyses of residential case-control studies to estimate the number of lung cancer deaths in 1999 that may have been associated with residential radon exposure. The analysis considers the variability of indoor radon exposure in France and allows the quantification of uncertainties related to each of the exposure–response relations.

## Materials and Methods

Following the risk assessment procedure proposed by the Covello and Merkhofer (1993) and the NRC (1983), we estimated the number of lung cancer deaths due to indoor radon exposure in France in a four-stage process: identification of the population, choice of the exposure–response relations, radon exposure assessment, and characterization of lung cancer risk.

### Identification of the population

We estimated the number of lung cancer deaths attributable to indoor radon exposure for the entire French population for the year 1999. The population data, including the age, sex, and geographic distributions, come from the 1999 census, conducted by the French National Institute of Statistics and Economics (INSEE 2000). In 1999, the total French population consisted of 61,889,304 people. The sex ratio was close to 1; 24% were < 20 years of age, 43% were 20–49 years, 21% were 50–69 years, and 12% ≥ 70 years.

Data on lung cancer deaths come from the French Institute of Health and Medical Research (INSERM; Paris, France), the center responsible for compiling national mortality statistics. In 1999, 25,134 people died of lung cancer in France—20,823 men and 4,321 women.

Data about tobacco consumption in France are sparse. Information on the proportion of ever-smokers in the general population comes from studies conducted in the 1990s by the French Research and Information Institute for Health Economics (Hill and Laplanche 2003). On average, 65% of men and 31% of women were current or ex-smokers at that time. Although the proportion of men who smoke increases with age (from approximately 55% of those 16–39 years of age to approximately 70% of those ≥ 40 years), the proportion among women decreases with age (from approximately 47% of women 16–39 years of age to < 30% of those ≥ 40 years) (Hill and Laplanche 2003).

### Choice of exposure–response relations

We used the exposure–response relations determined in the main epidemiologic studies of radon: the joint analysis of 11 cohorts of miners (BEIR 1999; Lubin et al. 1994), the joint analysis of French and Czech uranium miners (Tirmarche et al. 2003), and the two available joint analyses of case-control studies (Darby et al. 2005; Lubin 2003). They are briefly described below.

#### Joint analysis of 11 cohorts of miners

The initial combined analysis of 11 cohorts of miners was published in 1994 (Lubin et al. 1994). In 1999, the BEIR committee reanalyzed these data and derived two models for lung cancer risk from radon exposure (BEIR 1999). Both models express the risk as the excess relative risk (ERR), which they define as the multiplicative increment in the excess lung cancer risk due to radon above background levels. The ERR is a linear function of cumulative exposure and varies according to time, since exposure, age, and either exposure-age-duration (EAD model) or exposure-age-concentration (EAC model). In either case, its form is as follows:


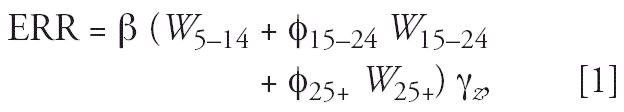


where β is the slope parameter of the exposure–response relation; *W*_5–14_, *W*_15–24_ and *W*_25+_ are the exposure cumulated 5–14, 15–24, and ≥ 25 years earlier, respectively; and φ_5–14_ (set at unity), φ_15–24_, and φ_25+_ are the associated parameters. The parameter γ*_z_* represents the modifying effect of either the duration of radon exposure (in the EAD model) or radon concentration (in the EAC model). Details of this model and its implementation are presented elsewhere (BEIR 1999).

During the 20th century, active smoking became the leading cause of lung cancer (Peto et al. 1992), and recent studies conclude that approximately 90% of lung cancers occur among smokers (Beckett 1993; Hill 1998; Peto et al. 1992, 2000; Pierce et al. 1992). The importance of the lung cancer risk caused by active smoking makes it essential to quantify the possible interaction between tobacco and radon. In 1999, the BEIR committee conducted such an analysis of the five cohorts of miners with adequate smoking data and found that lung cancer risk due to radon exposure differs according to smoking status (BEIR 1999). The available data, although not sufficient for detailed quantification of this interaction, indicated that it was submultiplicative. Despite the sparseness of data, the committee proposed using a factor of 2 for the ERR for nonsmokers and of 0.9 for smokers (BEIR 1999). We used these interaction factors to quantify the risk of lung cancer associated with indoor radon exposure separately for ever- and never-smokers.

#### Joint analysis of French and Czech uranium miners

In 2003, results from a European research project became available (Tirmarche et al. 2003). The analysis relied especially on the updated French and Czech cohorts of uranium miners, which included > 10,000 miners exposed to low levels of radon. These data provide an excellent basis for quantifying the risks associated with chronic radiation exposure at a relatively low dose rate. The average duration of follow-up exceeded that in the joint analysis of 11 cohorts of miners (24 years compared with 15). Inclusion criteria made it possible to focus on miners with high-quality exposure assessments and to quantify the exposure–response relation. The ERR increased with cumulative radon exposure and decreased as age at exposure and time since exposure increased. The model derived from this joint analysis [Franco Czech (FCZ) model] is a linear model that considers the modifying effects of age at median exposure, as well as time since median exposure. It is expressed as follows:


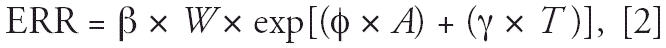


where β is the slope parameter of the exposure–response relation, and *W* is exposure cumulated until 5 years earlier. Variables *A* and *T* represent age at median exposure and time since median exposure, respectively, and φ and γ represent the associated parameters. Details of this model and its implementation are presented elsewhere (Tirmarche et al. 2003).

#### Joint analysis of residential case-control studies in North America

In 2003, Lubin reported the results of a joint analysis of residential data from seven North American case-control studies (Lubin 2003) conducted in Winnipeg, Canada, New Jersey, Missouri, Iowa, Connecticut, and Utah. The analysis included 4,081 cases of lung cancer (2,766 women and 1,315 men) and 5,281 controls. He estimated the relation between lung cancer risk and domestic radon exposure using a linear model:


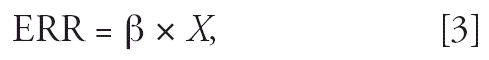


where β is the slope parameter of the exposure–response relation, and *X* is the mean radon concentration in the exposure time window (ETW), defined as 5–30 years before study enrollment. This ETW is the period during which radon is assumed to exert its most direct influence on lung cancer risk.

Lubin (2003) argued that the duration of the ETW covered by radon measurements must be increased to reduce uncertainty and improve the accuracy of exposure assessment. Consequently, he derived several exposure–response relations according to the duration of residential measurements. In our study, we retain two of these estimates: the first is based on individuals for whom domestic exposure to radon was measured at least once during the ETW (Lubin1) and the second on individuals with measurements that cover the entire ETW (Lubin25). Details of these models and their implementation are presented elsewhere (Lubin 2003).

#### Joint analysis of residential case-control studies in Europe

In 2005, the *British Medical Journal* (*BMJ*) published a joint analysis of 13 European case-control studies (Darby et al. 2005), including 7,148 cases of lung cancers and 14,208 controls. The authors estimated the relation between lung cancer risk and domestic radon exposure with this linear model:


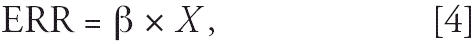


where β is the slope parameter of the exposure–response relation, and *X* is the mean radon concentration in the homes inhabited during the 5- to 34-year period before study enrollment. The estimated excess lung cancer risk is adjusted for study, age, sex, area of residence, and smoking status. These results are consistent with those from the miners’ studies and the North American joint analysis (Lubin 2003). Details of this model and its implementation are presented elsewhere (Darby et al. 2005).

### Radon exposure assessment

In 1982, the Institute for Radiological Protection and Nuclear Safety (IRSN), in collaboration with the French Ministry of Health, began a national radon measurement campaign, which has taken a total of 12,261 measurements (Billon et al. 2005; Gambard et al. 2000). These served as our source to assess the distribution of indoor radon concentrations in each department (French administrative entities approximately 5,000 km^2^ in area). The principal objectives of this campaign were to identify radon-prone areas in France, estimate the percentage of dwellings with measurements above action levels, and investigate factors affecting radon concentration. Dividing each department into grids of 36–49 km^2^ (depending on the department area) ensured homogeneous geographic distribution of the measurements. Grids that included municipalities with > 1,500 inhabitants had a second measurement taken in a different location. Volunteers were mainly recruited through contacts in the local governments, which placed and collected the radon detectors. Kodalpha LR 115 detectors (Dosirad Company, Lognes, France) measured indoor radon for 2 months.

The 12,261 indoor radon measurements yielded an arithmetic mean (± SD) concentration of 89 Bq/m^3^ ± 162 Bq/m^3^ and a median value of 55 Bq/m^3^. Indoor radon measurements ranged from 1 to 4,964 Bq/m^3^. The geometric mean (geometric SD) was 53 Bq/m^3^ (2 Bq/m^3^). After correcting for season of measurement, the arithmetic mean was 87 Bq/m^3^. The distribution of indoor radon measurements appears log normal.

Because indoor radon concentrations varied substantially between and within departments (Billon et al. 2005), we considered each department separately in the risk assessment to take the interdepartmental variations into account. We also used decile stratifications of each departmental distribution for the variations within departments.

**Figure 1:**
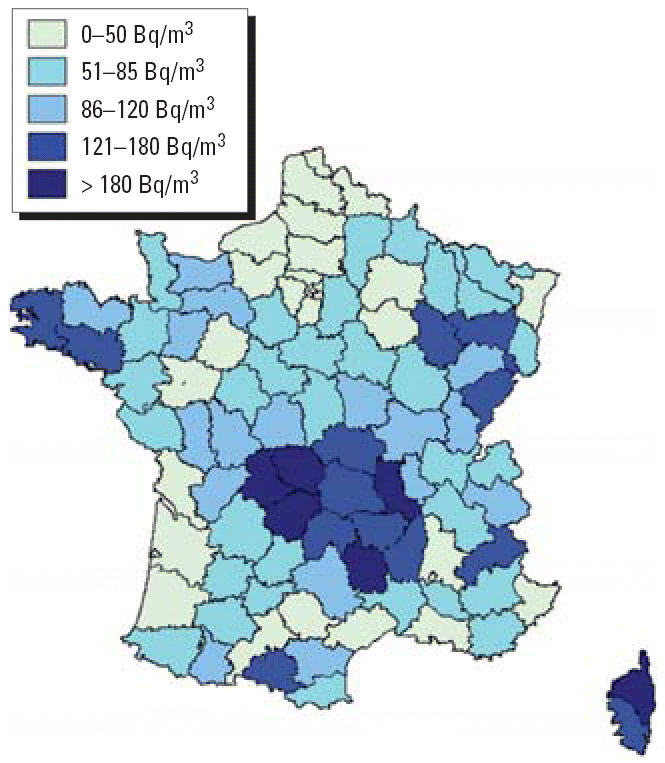
Average indoor radon concentration by department in France (Gambard et al. 2000).

Several studies from different countries have examined the influence of various factors on indoor radon concentrations (Arvela 1995; BEIR 1999; Pinel et al. 1995). Indoor radon measurements vary with the seasons; they are highest in winter and lowest in summer. Because radon concentrations were measured in France for 2-month sampling periods, correction for season was necessary to estimate annual concentration levels. Baysson et al. (2003) applied the model developed by Pinel et al. (1995) to the French database of indoor radon measurements to obtain seasonal correction factors for estimating radon exposure in dwellings in France. The calculation of the seasonal correction factors was made in three steps: first, the geometric means of monthly radon concentrations were estimated; second, a sine–cosine curve was fitted to these estimates; and third, the seasonal correction factors were derived from these sine–cosine estimates and from the measurement duration. We used these seasonal correction factors to estimate the distribution of indoor radon exposure by department (Figure 1).

### Characterization of lung cancer risk

Estimation of the number of lung cancer deaths attributable to indoor radon exposure requires combining several sets of data: the exposure–response relation between radon exposure and lung cancer risk, number of lung cancer deaths in France in 1999, percentage of smokers, and estimates of indoor radon exposure in France.

Application of the exposure–response relations mentioned above necessitates knowledge of the number of spontaneous deaths from lung cancer (i.e., apart from radon exposure). Most of the 25,134 lung cancer deaths observed in 1999 were probably due to smoking, some to indoor radon alone, others to the interaction between smoking and indoor radon, and the remainder to such other risk factors as air pollution and occupational exposure [Agence Française de Sécurité Sanitaire Environnementale (AFSSE) 2004; Darby et al. 2001]. To estimate the number of lung cancer deaths attributable to indoor radon exposure, we applied the data described above to the following formula:


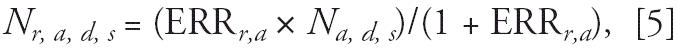


where *N_r, a, d, s_* is the number of lung cancer deaths due to indoor radon exposure at age *a*, in department *d* and for sex *s*. ERR*_r,a_* is the excess relative risk for age *a*, and radon exposure *r. N_a, d, s_* is the total number of lung cancer deaths at age *a* in department *d* and for sex *s*. Calculations were carried out by age, sex, and department.

### Uncertainty analysis

In an uncertainty analysis, based on all the data described above, we considered the uncertainties for each exposure–response relation and for the average indoor radon exposure by department according to its overall statistical distribution. We also took into account the variability of indoor radon exposure within each department. An uncertainty interval (UI) at the 5th and 95th percentiles represents the estimated impact of these uncertainties and variabilities (Greenland 2001), and dividing its 95th percentile by its 5th percentile provides an uncertainty coefficient. The uncertainty analysis relies on a Latin Hypercube approach (Iman and Conover 1980), with analyses for each department by Risk software (5,000 iterations) (Palisade Corporation 2001).

The key to Latin Hypercube sampling is stratification into equal intervals of the cumulative probability distribution of each input parameter (Iman and Conover 1980). The number of stratifications was set to equal the number of iterations performed. Randomly taking a sample from each stratification thus recreates the input probability distribution.

A two-step analysis considered the uncertainties associated with the exposure–response relation. In the first, we compared estimates from the risk models considered. That is, we considered six separate exposure–response relations. The second step consisted of quantifying uncertainties associated with each of the six exposure–response relations. We then approximated these six exposure–response relations and their confidence intervals to the more appropriate statistical distribution of the risk coefficients (BEIR 1999; Darby et al. 2005; Lubin 2003; Tirmarche et al. 2003). This step made it possible to determine a UI associated with the number of lung cancer deaths estimated according to each of the six exposure–response relations.

Despite the uncertainties concerning the interaction parameter between tobacco and radon proposed by BEIR (1999), no one has published a variance estimate for this coefficient. To reflect this uncertainty, we assumed that it varies by approximately 10% around the published value, according to a uniform distribution.

## Results

Table 1 shows the estimated number of lung cancer deaths attributable to indoor radon exposure in France, in view of the uncertainties about the exposure–response relation and the geographic variations of radon exposure. Depending on the risk model used, the total number of lung cancer deaths associated with indoor radon exposure in France for 1999 ranges from 543 (90% UI, 75–1,097) to 3,108 (90% UI, 2,996–3,221). The calculations suggest that of the 25,134 lung cancer deaths in France that year, from 2% (90% UI, 0.3–4.4%) to 12% (90% UI, 11–13%) may be attributed to indoor radon exposure. These estimates vary according to the exposure–response relation used in the risk assessment (Table 1). The Lubin1 model, from the joint analysis of the seven North American case-control studies (Lubin 2003), produced the fewest attributable deaths, the model derived from the French and Czech cohort of uranium miners (Tirmarche et al. 2003) the most. Table 1 shows that uncertainty coefficients based on models from the cohort of miners are lower than those based on models from joint analyses of general population case-control studies.

Table 2 reports the estimated number of lung cancer deaths attributable to indoor radon exposure in France in 1999, taking into account the interaction between tobacco and radon on the risk of lung cancer. Approximately three times more deaths occurred among smokers than among nonsmokers. The calculations suggest that the attributable percentage is low for smokers: of the 23,626 lung cancer deaths among smokers, from 8% (90% UI, 7–9%) to 11% (90% UI, 10–12%) may be attributable to indoor radon exposure, whereas from 36% (90% UI, 32–40) to 50% (90% UI, 46–55) of the 1,508 lung cancer deaths among nonsmokers may be so attributable.

**Figure 2:**
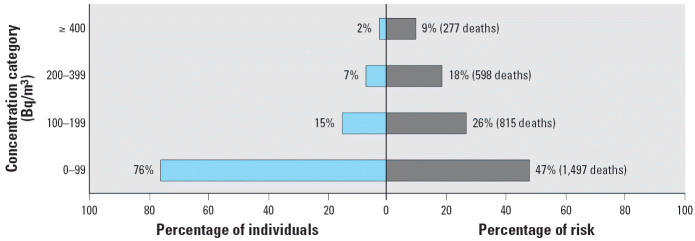
Proportion of individuals and deaths attributable to indoor radon exposure in France in 1999 according to the EAD model (BEIR 1999).

Although most of the radon-attributable lung cancer deaths in France may be due to exposure of < 200 Bq/m^3^ (Figure 2), 27% appear to occur among the 9% of the population exposed to concentrations > 200 Bq/m^3^.

## Discussion

In this study we estimate the number of lung cancer deaths in France in 1999 attributable to indoor radon exposure, according to the risk assessment method proposed by the NRC (Covello and Merkhofer 1993; NRC 1983). The consideration of several different exposure–response relations, which come from either cohorts of miners or residential case-control studies, allows us to compare estimates of attributable deaths based on various exposure–response relations. Data from miners makes it possible to investigate the interaction between tobacco and radon (BEIR 1999). We also considered the variability of domestic exposure to radon between and within French departments, as well as uncertainties associated with the exposure–response coefficients.

This risk assessment suggests that from 543 (90% UI, 75–1,097) to 3,108 (90% UI, 2,996–3,221) lung cancer deaths in France may be attributable to indoor radon exposure each year. Of the 25,134 lung cancer deaths in France in 1999, from 2% (90% UI, 0.3–4.4%) to 12% (90% UI, 11–13%) may be attributable to indoor radon exposure. These results must be interpreted according to the number of people in each exposure category. Thus, 47% of the estimated lung cancer deaths attributable to domestic radon exposure appear to occur among the 76% of the French population exposed to concentrations < 100 Bq/m^3^.

Deaths due to lung cancer attributable to indoor radon exposure can be considered premature because approximately half occur before the age of 70 years. We did not calculate the number of years of lost life, but given the long life expectancy in France, this figure may be quite high; management of the risk due to radon is clearly a major public health issue in France.

Previous risk assessments have estimated the number of lung cancer deaths attributable to indoor radon exposure, in particular, in the United States, the United Kingdom, France, and Canada (BEIR 1999; Brand et al. 2005; Darby et al. 2001; Pirard and Hubert 2001; U.S. EPA 2003) and concluded that 6–14% of all lung cancer deaths may be attributable to domestic radon exposure. Most of these studies used only the exposure–response relations based on the joint analysis of 11 international cohorts of miners recommended by the BEIR committee (BEIR 1999). Our analysis is the first to take into account several exposure–response relations in a single risk assessment. Because miners are generally exposed at higher levels than the general population, use of data from miners for assessment of general population risks clearly raises methodologic issues, especially those related to the extrapolation of risk from high to low exposure and the transposition of risk estimates from miners to the general population (BEIR 1999). Although the danger of radon is no longer subject to debate, the use of risk models based on occupational exposure to assess the lung cancer risk attributable to indoor radon exposure is rightfully still considered a problem.

Over the past two decades, many epidemiologic studies, mainly cohorts of miners and case-control studies in the general population, have estimated the lung cancer risk associated with radon exposure. In the present risk assessment we used different exposure–response relations from several of these studies and found that the number of lung cancer cases attributable to indoor radon appears relatively stable, regardless of the relation chosen. In this risk assessment, attributable risk estimates obtained from EAC and FCZ models appear conservative.

The uncertainty coefficient (> 9) is substantially higher for the results of the model based on the joint data analysis of seven North American case-control studies (Lubin 2003) than for those based on other models. This result is probably due to the lower statistical power of the former compared with the European joint analysis or the miner studies (BEIR 1999; Darby et al. 2005; Lubin 2003).

The two linear models recommended by the BEIR committee (1999) produce different numbers of attributable deaths: the calculations based on the EAD model yield 2,066 (90% UI, 1,934–2,203) attributable deaths and those based on the EAC model 2,913 (90% UI, 2,763–3,067). Previous assessments, especially those conducted in the United States and the United Kingdom, have already reported similar results (Darby et al. 2001; U.S. EPA 2003). These differences probably come from the model formulation. Whereas the EAD model considers the duration of exposure as a modifying factor of the exposure–response relation, the EAC model considers the concentration. Duration and concentration effects are both considered according to classes. All classes of duration effect are used to estimate the risk attributable to indoor radon exposure using the EAD model; however, only the first class of concentration effect is used with the EAC model. Both models, however, have the same quality of adjustment to the data from miners, which is why the BEIR committee (BEIR 1999) and Lubin et al. (1994) could not choose between them. Use of these two exposure–response relations in this risk assessment illustrates that the adequacy of the model formulation relative to the study population appears to be at least as important as the quality of adjustment of the model to the epidemiologic data.

### Risk extrapolation from miners to the general population

Because cumulative exposure and exposure rates were generally much higher in mines than in homes, using exposure–response relations determined among studies of miners to assess the risk due to indoor radon exposure requires extrapolation. To date, all published studies use a no-threshold linear relation for this extrapolation (BEIR 1999; Brand et al. 2005; Darby et al. 2001; U.S. EPA 2003). Results from experimental and epidemiologic studies (BEIR 1999; Brenner et al. 2003; Tirmarche et al. 2003) justify this assumption. Exposure–response relations determined from studies of miners and in case-control studies among the general population are consistent with a linear relation without any evidence of a threshold at low exposures (Darby et al. 2005; Tirmarche et al. 2003). Moreover, the attributable risk of lung cancer due to indoor radon exposure assessed in our analysis on the basis of the joint analyses of indoor case-control studies is compatible with the risk observed in studies of miners (Darby et al. 2005; Lubin 2003). Recent joint analyses of case-control studies in general populations validate results from studies of miners for levels of exposure observed in the general population (Darby et al. 2005; Lubin 2003).

The analysis of data from miners shows that age at exposure and time since exposure significantly modify the exposure–response relation; for the same cumulative exposure, longer exposure is associated with higher risks (BEIR 1999). This is an inverse dose rate effect. The structure of the models developed in these studies of miners prevents consideration of this effect in the risk assessment for the general population: the lower dose and exposure rate categories in the BEIR models (BEIR 1999) rely on very few data points, and the exposure in nearly all French homes falls into the lowest dose rate class considered in the BEIR models. Although cumulative exposures for general populations are close to those estimated in studies of miners, the dose rate in mines is from 100 to 1,000 times higher than that observed in general populations. Thus, if the inverse dose rate effect exists, the risk of lung cancer is underestimated in our risk assessment. Nevertheless, an analysis published by Lubin et al. (1997) suggests that the inverse dose rate effect is smaller when cumulative exposures are low. The recent joint analysis of French and Czech miners (Tirmarche et al. 2003) confirmed this: low annual exposure over a long duration characterizes both cohorts, and an inverse dose rate effect at these low doses was not observed.

### Risk transposition from miners to the general population

Using exposure–response relations determined among miners to assess the risk in the general population also requires their transposition and underlines some of the differences between these two populations, including sex and age distribution (BEIR 1999). Miners are men of working age, whereas the general population comprises men and women of all ages. Moreover, various physical and biological factors (e.g., ventilatory flow, type of breathing, tracheobronchial configuration, individual size) can modify exposure and risk. Because of the lack of data, we had to assume that the risk associated with the domestic exposure to radon is close to that observed among miners, regardless of sex and age at exposure. These are common assumptions in risk assessments (BEIR 1999; Brand et al. 2005; Darby et al. 2001; U.S. EPA 2003). Nevertheless, methodologic issues prevent case-control studies from quantifying the variation in risk according to factors such as time since exposure or dose rate.

### Interaction with tobacco

The major risk factor for lung cancer is tobacco. According to Peto et al. (2000), approximately 90% of lung cancer deaths occur among smokers. Since the early 1980s, studies of miners have sought to quantify the possible interaction between radon and tobacco in the risk of lung cancer. Currently, only five cohorts of miners—China, Colorado, Newfoundland, Malmberger, and New Mexico (BEIR 1999)—have collected smoking data sufficient to allow an approximate if imprecise quantification of this interaction. Thus, at present, the only source of data for assessing this risk is the recommendations of the BEIR committee (BEIR 1999). Nevertheless, our results show that consideration of the interaction between radon and tobacco does not significantly affect the estimates. Thus, the EAD model calculates the attributable percentage of lung cancer at 9% (90% UI, 8–10%) when it considers the interaction between tobacco and indoor radon exposure and at 8% (90% UI, 8–9%) when it does not.

Uncertainties about the quantification of the interaction, as well as the lack of tobacco consumption data in France, make it difficult to take this interaction into account in lung cancer risk assessment. ung cancer due to indoor radon exposure assessed in our analysis on the basis of the joint analyses of indoor case-control studies is compatible with the risk observed in studies of miners (Darby et al. 2005; Lubin 2003). Recent joint analyses of case-control studies in general populations validate results from studies of miners for levels of exposure observed in the general population (Darby et al. 2005; Lubin 2003).

The analysis of data from miners shows that age at exposure and time since exposure significantly modify the exposure–response relation; for the same cumulative exposure, longer exposure is associated with higher risks (BEIR 1999). This is an inverse dose rate effect. The structure of the models developed in these studies of miners prevents consideration of this effect in the risk assessment for the general population: the lower dose and exposure rate categories in the BEIR models (BEIR 1999) rely on very few data points, and the exposure in nearly all French homes falls into the lowest dose rate class considered in the BEIR models. Although cumulative exposures for general populations are close to those estimated in studies of miners, the dose rate in mines is from 100 to 1,000 times higher than that observed in general populations. Thus, if the inverse dose rate effect exists, the risk of lung cancer is underestimated in our risk assessment. Nevertheless, an analysis published by Lubin et al. (1997) suggests that the inverse dose rate effect is smaller when cumulative exposures are low. The recent joint analysis of French and Czech miners (Tirmarche et al. 2003) confirmed this: low annual exposure over a long duration characterizes both cohorts, and an inverse dose rate effect at these low doses was not observed.

### Risk transposition from miners to the general population

Using exposure–response relations determined among miners to assess the risk in the general population also requires their transposition and underlines some of the differences between these two populations, including sex and age distribution (BEIR 1999). Miners are men of working age, whereas the general population comprises men and women of all ages. Moreover, various physical and biological factors (e.g., ventilatory flow, type of breathing, tracheobronchial configuration, individual size) can modify exposure and risk. Because of the lack of data, we had to assume that the risk associated with the domestic exposure to radon is close to that observed among miners, regardless of sex and age at exposure. These are common assumptions in risk assessments (BEIR 1999; Brand et al. 2005; Darby et al. 2001; U.S. EPA 2003). Nevertheless, methodologic issues prevent case-control studies from quantifying the variation in risk according to factors such as time since exposure or dose rate.

### Interaction with tobacco

The major risk factor for lung cancer is tobacco. According to Peto et al. (2000), approximately 90% of lung cancer deaths occur among smokers. Since the early 1980s, studies of miners have sought to quantify the possible interaction between radon and tobacco in the risk of lung cancer. Currently, only five cohorts of miners—China, Colorado, Newfoundland, Malmberger, and New Mexico (BEIR 1999)—have collected smoking data sufficient to allow an approximate if imprecise quantification of this interaction. Thus, at present, the only source of data for assessing this risk is the recommendations of the BEIR committee (BEIR 1999). Nevertheless, our results show that consideration of the interaction between radon and tobacco does not significantly affect the estimates. Thus, the EAD model calculates the attributable percentage of lung cancer at 9% (90% UI, 8–10%) when it considers the interaction between tobacco and indoor radon exposure and at 8% (90% UI, 8–9%) when it does not.

Uncertainties about the quantification of the interaction, as well as the lack of tobacco consumption data in France, make it difficult to take this interaction into account in lung cancer risk assessment. ung cancer due to indoor radon exposure assessed in our analysis on the basis of the joint analyses of indoor case-control studies is compatible with the risk observed in studies of miners (Darby et al. 2005; Lubin 2003). Recent joint analyses of case-control studies in general populations validate results from studies of miners for levels of exposure observed in the general population (Darby et al. 2005; Lubin 2003).

The analysis of data from miners shows that age at exposure and time since exposure significantly modify the exposure–response relation; for the same cumulative exposure, longer exposure is associated with higher risks (BEIR 1999). This is an inverse dose rate effect. The structure of the models developed in these studies of miners prevents consideration of this effect in the risk assessment for the general population: the lower dose and exposure rate categories in the BEIR models (BEIR 1999) rely on very few data points, and the exposure in nearly all French homes falls into the lowest dose rate class considered in the BEIR models. Although cumulative exposures for general populations are close to those estimated in studies of miners, the dose rate in mines is from 100 to 1,000 times higher than that observed in general populations. Thus, if the inverse dose rate effect exists, the risk of lung cancer is underestimated in our risk assessment. Nevertheless, an analysis published by Lubin et al. (1997) suggests that the inverse dose rate effect is smaller when cumulative exposures are low. The recent joint analysis of French and Czech miners (Tirmarche et al. 2003) confirmed this: low annual exposure over a long duration characterizes both cohorts, and an inverse dose rate effect at these low doses was not observed.

### Risk transposition from miners to the general population

Using exposure–response relations determined among miners to assess the risk in the general population also requires their transposition and underlines some of the differences between these two populations, including sex and age distribution (BEIR 1999). Miners are men of working age, whereas the general population comprises men and women of all ages. Moreover, various physical and biological factors (e.g., ventilatory flow, type of breathing, tracheobronchial configuration, individual size) can modify exposure and risk. Because of the lack of data, we had to assume that the risk associated with the domestic exposure to radon is close to that observed among miners, regardless of sex and age at exposure. These are common assumptions in risk assessments (BEIR 1999; Brand et al. 2005; Darby et al. 2001; U.S. EPA 2003). Nevertheless, methodologic issues prevent case-control studies from quantifying the variation in risk according to factors such as time since exposure or dose rate.

### Interaction with tobacco

The major risk factor for lung cancer is tobacco. According to Peto et al. (2000), approximately 90% of lung cancer deaths occur among smokers. Since the early 1980s, studies of miners have sought to quantify the possible interaction between radon and tobacco in the risk of lung cancer. Currently, only five cohorts of miners—China, Colorado, Newfoundland, Malmberger, and New Mexico (BEIR 1999)—have collected smoking data sufficient to allow an approximate if imprecise quantification of this interaction. Thus, at present, the only source of data for assessing this risk is the recommendations of the BEIR committee (BEIR 1999). Nevertheless, our results show that consideration of the interaction between radon and tobacco does not significantly affect the estimates. Thus, the EAD model calculates the attributable percentage of lung cancer at 9% (90% UI, 8–10%) when it considers the interaction between tobacco and indoor radon exposure and at 8% (90% UI, 8–9%) when it does not.

Uncertainties about the quantification of the interaction, as well as the lack of tobacco consumption data in France, make it difficult to take this interaction into account in lung cancer risk assessment. Current epidemiologic studies are working to improve the quantification of this interaction between tobacco and radon (Tirmarche et al. 2003). Our knowledge of tobacco consumption in France by sex, age, and department (Hill and Laplanche 2003) has gaps that limit our computations to the ZEAT scale (French administrative entities approximately 60,000 km^2^ in area). We were not able to consider the variability of indoor radon concentration within departments, which may underestimate the dispersion of the attributable risk.

### Uncertainties and variability of the exposure

Numerous uncertainties are associated with the estimation of the indoor radon exposure of the French population. Most come from measurement errors and localization choices. Unfortunately, impact of these uncertainties on the attributable risk calculations is very difficult to quantify. However, three main arguments are in favor of a limited impact: *a*) the measurement method used during all the national radon measurement campaign is standardized and has been normalized (Association Française de Normalisation 2004); *b*) the exposure database is quite large (> 12,000 points of measurement) and represents with reasonable precision the geographic distribution of indoor radon exposure; *c*) moreover, an intercomparison of passive radon detectors (Kreienbrock et al. 1999) concluded that exposure assessments for epidemiologic studies based on radon measurements using passive devices were reasonable.

In addition to radon, thoron is another natural radioactive gas entering homes. The open detectors record alpha particles originating from radon, thoron, and their decay products in the ambient atmosphere. The Kodalpha LR115 detector does not allow the elimination of thoron, therefore giving misleading information concerning radon concentration. Thoron has a half-life of 55 sec, but radon has a half-life of 4 days; therefore, the very short half-life of thoron prevents it from diffusing very far before it decays to thoron progeny. Compared with radon, exposure from thoron is inconsequential in homes.

The indoor radon measurements taken in France since the early 1980s reveal strong variability in radon concentrations (Gambard et al. 2000). In our study, we considered this variability with decile stratifications of the distribution for each department. The variations appear to depend mainly on building type, the quality of its ventilation, local geology, season, and activities of the occupants. The large number of measurements in France—an average of 128 measurements per department—have made it possible to estimate the variability of radon concentrations and thus of indoor radon exposures.

## Conclusion

This study is the first assessment of the nationwide risk associated with residential radon exposure in France. Such an assessment is a topic of concern to the European Community and the World Health Organization. This lung cancer risk assessment is the first to consider simultaneously different exposure–response relations and thus to compare the risks estimated by each. When we take into account uncertainties related to the exposure–response relation and geographic variations in radon exposure, we find that the total number of lung cancer deaths in 1999 attributable to indoor radon exposure in France ranges from 1,234 (90% UI, 593–2,156) to 3,108 (90% UI, 2,996–3,221). Of the 25,134 lung cancer deaths in France during 1999, indoor radon probably caused 5–12%.

## Figures and Tables

**Table 1:** Estimates of lung cancer deaths attributable to indoor radon exposure in France in 1999 according to different exposure–response relations.

	No. of lung cancer deaths attributable to indoor radon								
				Percentiles				Attributable percentage	
Dose–response relationships	Mean ± SD	UI (90%)^a^	Mode	10th	50th	90th	Dispersion	Mean	UI (90%)^a^
Studies of miners									
EAD^b^	2,066 ± 82	1,934–2,203	2,001	1,962	2,064	2,171	1.14	8	8.0–9.0
EAC^c^	2,913 ± 92	2,763–3,067	2,834	2,795	2,912	3,032	1.11	12	11.0–12.0
FCZ^d^	3,108 ± 68	2,996–3,221	3,028	3,020	3,107	3,195	1.08	12	12.0–13.0
Indoor studies									
Lubin1^e^	543 ± 314	75–1,097	485	139	519	970	14.60	2	0.3–4.4
Lubin25^f^	2,642 ± 1,396	518–5,121	2,982	920	2,856	4,671	9.90	11	2.1–20.0
Darby	1,234 ± 492	593–2,156	995	688	1,151	1,884	3.64	5	2.4– 9.0

a UIs from the uncertainty analysis.

b EAD model (BEIR 1999).

c EAC model (BEIR 1999).

d Risk model from the European research project concerning the French and Czech cohort of uranium miners (Tirmarche et al. 2003).

e Risk model from the joint analysis of the seven North American case-control studies restricted to subjects with some radon measurments within the ETW of 25 years (Lubin 2003).

f Risk model from the joint analysis of the seven North American case-control studies restricted to individuals for whom measurements covered the whole ETW (Lubin 2003).

**Table 2:** Estimates of predicted lung cancer deaths attributable to indoor radon exposure in France in 1999, considering the interaction between tobacco and radon.

	No. of lung cancer deaths attributable to indoor radon								
				Percentiles				Attributable percentage	
Dose–response relationships	Mean ± SD	UI (90%)^a^	Mode	10th	50th	90th	Dispersion	Mean	UI (90%)^a^
EAD^b^									
Smokers	1,819 ± 122	1,624–2,019	1,718	1,660	1,818	1,980	1.24	8	7–9
Nonsmokers	541 ± 33	489–597	521	500	541	584	1.22	36	32–40
Total	2,361	2,112–2,616						9	8–10
EAC^c^									
Smokers	2,578 ± 155	2,329–2,830	2,473	2,374	2,578	2,782	1.22	11	10–12
Nonsmokers	759 ± 37	700–822	738	712	759	807	1.17	50	46–55
Total	3,337	3,029–3,652						13	12–15

a UIs obtained from the uncertainty analysis.

b EAD model (BEIR 1999), considering the interaction between tobacco and radon.

c EAC model (BEIR 1999), considering the interaction between tobacco and radon.
